# Chromogranin A, a significant prognostic factor in small cell lung cancer

**DOI:** 10.1038/sj.bjc.6690745

**Published:** 1999-10

**Authors:** L Drivsholm, L I Paloheimo, K Østerlind

**Affiliations:** Departments of Oncology, Clinical Chemistry, Rigshospitalet, Copenhagen, Denmark; Medical Department F, Hillerød Sygehus, Denmark

**Keywords:** chromogranin A, small cell lung cancer, tumour marker, prognostic factors

## Abstract

Chromogranin A (CgA) is a protein present in neuroendocrine vesicles. Small cell lung cancer (SCLC) is considered a neuroendocrine tumour. It is possible to demonstrate CgA expression in SCLC by immunohistochemical methods. Since CgA is released to the circulation it might also work as a clinical tumour marker. We used a newly developed two-site enzyme-linked immunosorbent assay for CgA in plasma from 150 newly diagnosed patients with SCLC. Follow-up was for a minimum of 5 years. Thirty-seven per cent of the patients had elevated pretreatment values and the values were significantly related to stage of disease. Multivariable analysis by Cox's proportional hazard model including nine known prognostic factors disclosed performance status as the most influential prognostic factor followed by stage of disease, CgA and LDH. A simple prognostic index (PI) could be established based on these four pretreatment features. In this way the patients could be separated into three groups with significant different prognosis. The median survival and 95% confidence intervals for the three groups were as follows: 424 days (311–537), 360 days (261–459) and 174 days (105–243). © 1999 Cancer Research Campaign


British Journal of Cancer (1999) 81(4), 667–671
© 1999 Cancer Research Campaign
Article no. bjoc.1999.0745

### Chromogranin A, a significant prognostic factor in small cell lung cancer

**L Drivsholm[1], LI Paloheimo[2] and K Østerlind[3]**

Departments of [1]Oncology and [2]Clinical Chemistry, Rigshospitalet, Copenhagen, Denmark; [3]Medical Department F, Hillerød Sygehus, Denmark

**Summary** Chromogranin A (CgA) is a protein present in neuroendocrine vesicles. Small cell lung cancer (SCLC) is considered a neuroendocrine tumour. It is possible to demonstrate CgA expression in SCLC by immunohistochemical methods. Since CgA is released to the circulation it might also work as a clinical tumour marker. We used a newly developed two-site enzyme-linked immunosorbent assay for CgA in plasma from 150 newly diagnosed patients with SCLC. Follow-up was for a minimum of 5 years. Thirty-seven per cent of the patients had elevated pretreatment values and the values were significantly related to stage of disease. Multivariable analysis by Cox's proportional hazard model including nine known prognostic factors disclosed performance status as the most influential prognostic factor followed by stage of disease, CgA and LDH. A simple prognostic index (PI) could be established based on these four pretreatment features. In this way the patients could be separated into three groups with significant different prognosis. The median survival and 95% confidence intervals for the three groups were as follows: 424 days (311–537), 360 days (261–459) and 174 days (105–243). © 1999 Cancer Research Campaign

**Keywords:** chromogranin A; small cell lung cancer; tumour marker; prognostic factors

Several possible tumour markers from the blood of patients with small cell lung cancer (SCLC) have been described (Ferrigno and Buccheri, 1995). The search for new ones continues in the hope of finding a marker which alone or in combination with other markers could be helpful in prognosis-estimation, staging or monitoring of treatment. Chromogranin A (CgA) is well described as a histochemical marker in SCLC (Rosa and Gerdes, 1994) but few results regarding blood values of CgA from patients with SCLC have been published (Sobol et al, 1986; Johnson et al, 1993). Chromogranin A is a 49 kDa glycoprotein, reported for the first time 30 years ago (Banks and Helle, 1965; Blaschko et al, 1967). The primary structure consists of 439 amino acids (Konecki et al, 1987); and its gene is located on chromosome 14 (Murray et al, 1987). It is found in the neurosecretory granules of normal and malignant neuroendocrine cells. CgA is released into the circulation via exocytosis from neuroendocrine storage vesicles. The role of CgA is not known precisely, but possible functions include intracellular regulation of the formation of granules, regulation of hormone secretion and function as a prohormone (Helle and Angeletti, 1994; Hendy et al, 1995; Iacangelo and Eiden, 1995).

The aim of this study was to evaluate the value of plasma CgA as a tumour marker in SCLC.

### MATERIALS AND METHODS

Plasma samples were obtained after informed consent from 150 consecutive patients referred to the four hospitals participating in the 'Copenhagen Lung Cancer Study Group' (Hirsch et al, 1994), in the period April 1989 to January 1991. All patients had no prior cancer and histologically confirmed SCLC except for 15 patients from whom only cytological material was available. Before treatment, the patients were classified as having limited or extensive disease (LD/ED) on the basis of clinical examination, chest X-ray, bone-marrow aspiration and biopsy from the iliac crest (unilateral: ten patients; bilateral: 130 patients; not done: ten patients), and ultrasound of the liver with biopsy, if possible, of suspect regions. LD was defined as disease confined to one hemithorax excluding proven malignant pleural effusion and chest wall metastases. Ipsilateral supraclavicular lymph nodes were included in the criteria of LD. Performance status (PS) was scored according to the WHO system. Various biochemical tests, including complete blood counts, plasma sodium, LDH, aspartate aminotransferase (AST) and alkaline phosphatase (AP) were done. The pretreatment characteristics for the patients are shown in Table 1. All samples including plasma for CgA analysis were collected before the initiation of chemotherapy. Patients were treated according to treatment protocols including combinations of platin analogues, podophyllotoxin derivatives, alkylating agents and vinca alkaloids (Hirsch et al, 1994). None of the protocols included either surgery or radiotherapy. Follow-up time for seven (8%) long time survivors was for a minimum of 5 years. Control subjects were 28 healthy persons.

Blood samples were collected into tubes containing 3.9 μmol ethylene-diaminetetraacetate (EDTA) per ml of blood and kept

**Table 1** Pretreatment characteristics in 150 patients with SCLC

| | LD | ED | LD + ED |
|---|---|---|---|
| Number of patients (%) | 75 (50%) | 75 (50%) | 150 (100%) |
| Number of males (%) | 45 (30%) | 47 (31%) | 92 (61%) |
| Median years of age (range) | 65 (35–75) | 62 (44–79) | 63 (35–79) |

*Received 5 November 1998*
*Revised 10 February 1999*
*Accepted 16 April 1999*

*Correspondence to:* L Drivsholm, Engbakken 36, DK-2830 Virum, Denmark

**Table 2** Median duration of survival: influence of nine pretreatment clinical features

| Variable | Score[a] | No. of patients examined | Median survival (weeks) | $P^b$ | TT[c] |
|---|---|---|---|---|---|
| Sex: male vs female | 0,1 | 92 58 | 36 48 | 0.0050 | – |
| Age (Y): Y ≤ 60 vs 60 < Y ≤ 70 vs Y > 70 | 0,1,2 | 62 79 9 | 48 41 21 | 0.0005 | 0.1227 |
| Disease stage: limited vs extensive | 0,1 | 75 75 | 55 35 | 0.0010 | – |
| Performance status: 0–1 vs 2 vs 3–4 | 0,1,2 | 117 22 11 | 46 29 21 | 0.0013 | 0.0005 |
| CgA: ≤ 1.1 vs > 1.1 nmol $l^{-1}$ | 0,1 | 94 56 | 49 28 | 0.0014 | – |
| Na: < 136 vs ≥ 136 nmol $l^{-1}$ | 1,0 | 40 102 | 31 45 | 0.0280 | – |
| AST: ≤ 40 vs > 40 U $l^{-1}$ | 0,1 | 122 23 | 46 20 | 0.0029 | – |
| LDH: ≤ 450 vs 451–900 vs > 900 U $l^{-1}$ | 0,1,2 | 73 33 38 | 59 35 32 | 0.0012 | 0.0003 |
| AP: ≤ 275 vs 276–550 vs > 550 U/$l^{-1}$ | 0,1,2 | 92 31 20 | 46 38 27 | 0.0049 | 0.0014 |

[a]score used in Cox analysis (Table 4); [b]log rank; [c]TT: test for trend (log-rank).

**Table 3** Pretreatment chromogranin A values in nmol $l^{-1}$

| | *n* | Median | Range | 2p | Pct.elevated[a] |
|---|---|---|---|---|---|
| LD | 75 | 0.85 | 0.30–6.34 | 0.039 | 27% |
| ED | 75 | 0.97 | 0.25–9.08 | | 48% |

[a]Cut-off: 1.10 nmol $l^{-1}$.

**Table 4** Prognostic factors in SCLC based on Cox regression analysis of 144 patients

| Variable | Coefficient | SE | *P* | RR | 95% CI |
|---|---|---|---|---|---|
| Performance status | 0.4225 | 0.1463 | 0.0039 | 1.53 | (1.14–2.04) |
| Disease stage | 0.4553 | 0.1844 | 0.0135 | 1.58 | (1.09–2.28) |
| Chromogranin A | 0.4009 | 0.1851 | 0.0303 | 1.49 | (1.03–2.16) |
| LDH | 0.2525 | 0.1050 | 0.0162 | 1.29 | (1.04–1.59) |

cool. After centrifugation, plasma was stored at –80°C until assayed. Quantification of CgA in plasma was performed in duplicate by an enzyme-linked immunosorbent assay (Chromogranin A ELISA kits, code No. K 025, were kindly provided by Dako A/S, Glostrup, Denmark). Plasma samples from the patients were incubated simultaneously with peroxidase-conjugated anti-CgA in microtitre plates (96 wells) coated with anti-CgA. Polyclonal antibodies raised against a C-terminal fragment of CgA were used in the assay.

### Statistical analysis

Difference between values were tested for statistical significance by Mann–Whitney test, as the values were not normally distributed. Lifetable probabilities of overall survival were performed by the Kaplan–Meier method (Kaplan and Meier, 1958), and differences in survival between subgroups of patients were compared with the log-rank test (Mantel, 1996) plus the test for trend if there was a natural ordering of categories on the factor (Tarone, 1975). Overall length of survival was measured from the day chemotherapy was initiated. A $P$-value < 0.05 was considered significant. The prognostic impact of the following pretreatment variables was investigated by the use of Cox's proportional hazards multivariable regression model (Cox, 1972): sex, age, stage of disease, PS, LDH, sodium, AST and AP. Together with these variables, which have all previously been shown to be prognostic factors, CgA was included. All variables were divided into clinically meaningful groups (Table 2). All variables were included in the initial model and excluded step-wise based on the partial likelihood ratio test statistic.

Log minus log survival plots were made to check for proportionality between death hazards. Finally, an algorithm for prognostic categorization was created based on the regression coefficients in the final model.

The statistical procedures were done on a PC using the SPSS/7.0 (SPSS Inc., Chicago, IL, USA) software and the BMDP statistical software (UC Press, Berkeley, CA, USA).

### RESULTS

Median CgA-value from the healthy persons was 0.76 nmol $l^{-1}$ (range 0.47–1.10). Values above the 97.5th percentile – 1.10 nmol $l^{-1}$ – were considered to be abnormally high/positive. The CgA values for the 150 patients were as follows: median 0.89 nmol $l^{-1}$ (range and inter-quartile range: 0.25–9.08 and 0.59–1.39) and 37% had positive values. Patients with LD had a median CgA-value of 0.85 nmol $l^{-1}$ (range and inter-quartile range: 0.30–6.34 and 0.59–1.1) and with ED had a median CgA-value of 0.97 nmol $l^{-1}$ (range and inter-quartile range: 0.25–9.08 and 0.59–1.73). Patients with SCLC had significantly higher CgA than the control group ($P$ = 0.049). For patients with LD, 27% had elevated values, whereas 48% of patients with ED had elevated values. The concentrations in ED were significantly higher than in LD ($P$ = 0.039) and the group of healthy persons ($P$ = 0.017). The results are shown in Table 3. The CgA values were not related to specific patterns or number of metastases.

Patients with positive pretreatment CgA values lived significantly shorter ($P$ = 0.001) compared to patients with normal CgA values (Figure 1). The median survival and 95% confidence interval (CI) for patients with increased pretreatment CgA is 196 days (40–352) and 342 days (288–396) for patients with normal CgA values.

In order to study the influence of CgA beyond the first 30 days from the start of treatment (i.e. beyond the period of early death) another survival analysis was performed (Figure 2). There were 28 early deaths: progressive SCLC = 15, toxic death = six, 'unknown' = four and cardiovascular incidents = three. Pretreatment CgA maintains its negative impact on survival after the first 30 days ($P$ = 0.03).

In order to evaluate the prognostic influence of CgA compared to other known prognostic factors, a multivariate regression

 © 1999 Cancer Research Campaign

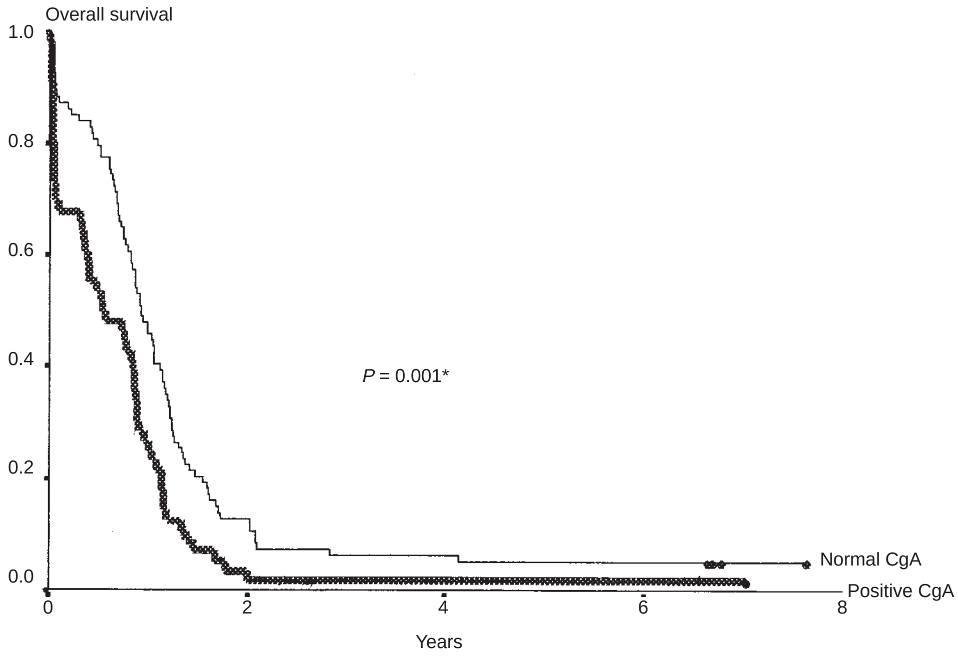

**Figure 1** Kaplan–Meier plot of the cumulative probability of survival for 150 patients with SCLC divided into two groups with normal and positive pretreatment CgA-values. Compared by log-rank test*. $P = 0.001$

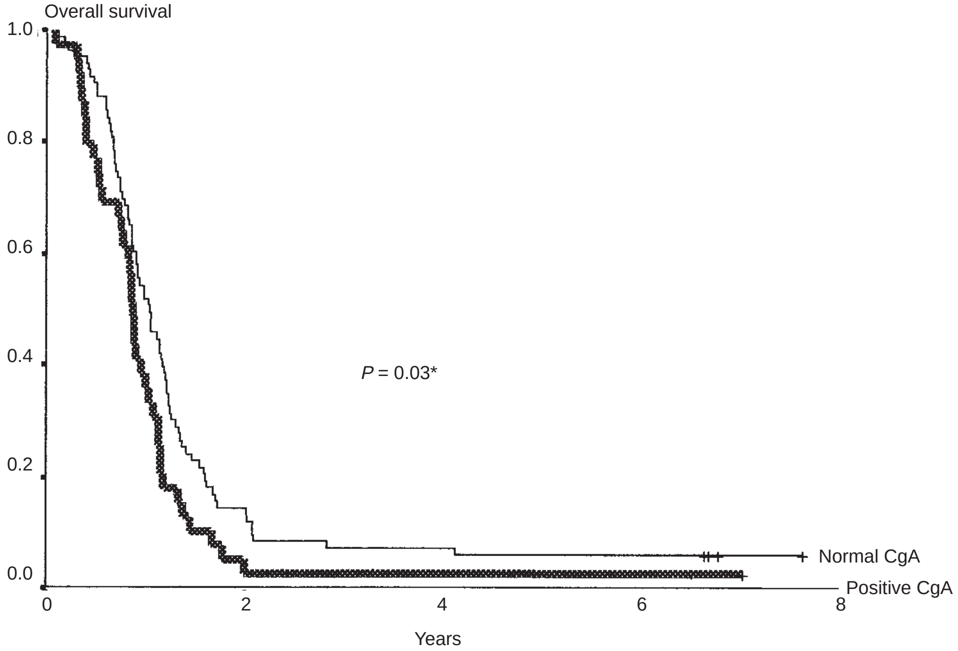

**Figure 2** Kaplan–Meier plot of the cumulative probability of survival for 122 patients with SCLC living more than 30 days (no 'early death') divided into two groups with normal and positive pretreatment CgA-values. Compared by log-rank test*. $P = 0.03$

analysis was performed. Results of univariate analyses are summarized in Table 2, which also shows the categorization (scoring) of the variables. The final Cox model is shown in Table 4. No significant interaction between the influences of P-CgA, PS, stage or S-LDH was found. The prognostic information of these four variables could be combined into a prognostic index such as $PI = 0.40 \times Z_{CgA} + 0.42 \times Z_{PS} + 0.46 \times Z_{stage} + 0.25 \times Z_{LDH}$ but we chose the approximation $PI = Z_{CgA} + Z_{PS} + Z_{stage} + 0.5 \times Z_{LDH}$, because it is much more handy in clinical practice. The resulting 11 prognostic strata were changed into three prognostic categories: good (PI = 0–0.5), intermediate (PI = 1.0–1.5) and poor (PI = 2.0–5.0) (Figure 3.).

### DISCUSSION

This study has shown that plasma CgA is increased to abnormal values in nearly 40% of patients with SCLC compared to healthy individuals. Patients with a large tumour burden (ED) have signifi-

*© 1999 Cancer Research Campaign* 

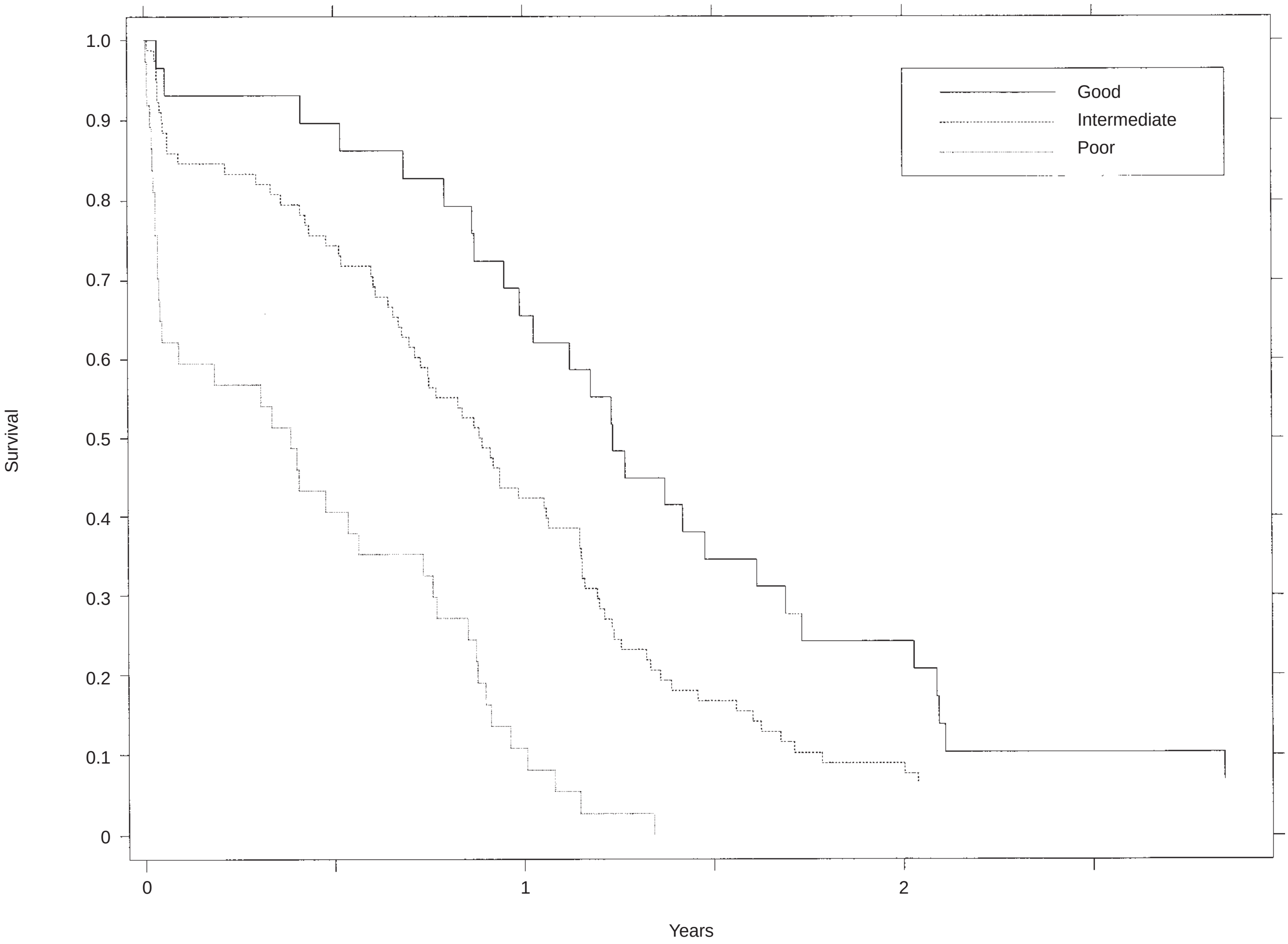

**Figure 3** Kaplan–Meier plot of the cumulative probability of survival for the three prognostic categories: good, intermediate and poor. Compared by log-rank test: $P < 0.00001$

cantly higher values compared to patients with a smaller tumour burden (LD). Survival is significantly worse for patients with elevated CgA values and CgA is a significant prognostic factor – also in multivariable analysis. To our knowledge, the only other Cox analysis including CgA in SCLC is Johnson et al (1993). Their model included NSE and albumin, and other cut-off levels were used in LDH, PS, CgA and sodium. Their final Cox model included NSE, PS and albumin, but this difference compared to our model may be accidental considering that 101 patients in their series, and 150 patients in our series, only enable identification of few (three to four) influential factors.

The purpose of the study was to evaluate the clinical usefulness of CgA as a prognostic factor and marker of disease activity – but not as a tool for screening. Therefore a 'reference group' of only 28 healthy individuals was accepted and tested. For healthy adults, the CgA values are not sex- or age-dependent (O'Connor and Deftos, 1987; Hsaio et al, 1991). Our study has not been able to confirm previous findings of elevated CgA in about 50% of patients with LD and in about 70% of patients with ED (Sobol et al, 1986; Johnson et al, 1993). Differences in analysis methods and material (serum instead of plasma) are the most plausible reasons for this. Chromogranin is probably a pro-hormone and several degradation products are known (Helle and Angetti, 1994). Precisely which is measured with the ELISA method needs clarification, and variability from one ELISA test to another could be a reason for lower values in this series.

Chromogranin A is a major constituent of catecholamine storage vesicles and it is released with epinephrine and norepinephrine during exocytosis. One could therefore expect physiological factors such as circadian cycle and stress to influence the concentrations of CgA. All plasma samples were taken in the morning before the patients got out of bed but not necessarily fasting. However, eating does not influence CgA (Takiyyuddin et al, 1990). Another factor of influence is the kidney function (Hsiao et al, 1990), but only one patient had a slightly increased pretreatment creatinine; and the elevated CgA value in this patient (3.67 nmol $l^{-1}$) is best explained by extensive disease stage.

Several parameters have been analysed for prognostic value in SCLC. The best described prognostic biochemical factor is LDH (Østerlind et al, 1986; Buccheri and Ferrigno, 1994). However, LDH is elevated in various malignant and non-malignant conditions including inflammation. Even though increased plasma CgA is not specific for SCLC, CgA is theoretically a much more specific marker for SCLC, since CgA apart from brain and adrenals only is present in tumour cells, whereas LDH is present in the cytoplasm of all cells in the body. It is therefore meaningful

© 1999 Cancer Research Campaign that plasma-CgA provides additional prognostic value, as confirmed in this investigation.

### ACKNOWLEDGEMENTS

This study was supported by grants from: 'Dagmar Marshall Foundation', 'Fritz, Georg and Marie Cecilie Glud Foundation', 'Danish Cancer League' and 'Foundation of 1986, Department of Oncology'.

### REFERENCES

Banks P and Helle K (1965) The release of protein from the stimulated adrenal medulla. *Biochem J* **97**: 40–41

Blaschko H, Comline RS, Schneider FH, Silver M and Smith AD (1967) Secretion of a chromaffin granule protein, chromogranin, from the adrenal gland after splanchnic stimulation. *Nature* **215**: 58–59

Buccheri G and Ferrigno D (1994) Prognostic factors in lung cancer: tables and comments. *Eur Respir J* **7**: 1350–1364

Cox DR (1972) Regression models and life-tables. *Stat Soc* **34**: 187–220

Ferrigno D and Buccheri G (1995) Clinical applications of serum markers for lung cancer. *Respir Med* **89**: 587–597

Helle KB and Angeletti RH (1994) Chromogranin A: a multipurpose prohormone? *Acta Physiol Scand* **152**: 1–10

Hendy GN, Bevan S, Mattei M-G and Mouland AJ (1995) Chromogranin A. *Clin Invest Med* **18**: 47–65

Hirsch FR, Dombernowsky P and Hansen HH (1994) Treatment of small cell lung cancer: the Copenhagen experience. *Anticancer Res* **14**: 317–320

Hsiao RJ, Parmer RJ, Takiyyuddin MA and O'Connor DT (1991) Chromogranin A storage and secretion: sensitivity and specificity for the diagnosis of pheochromocytoma. *Medicine* **70**: 33–45

Hsiao RJ, Mezger MS and O'Connor DT (1990) Chromogranin A in uremia: progressive retention of immunoreactive fragments. *Kidney Int* **37**: 955–964

Iacangelo AL and Eiden LE (1995) Chromogranin A: current status as a precursor for bioactive peptides and a granulogenic/sorting factor in the regulated secretory pathway. *Regul Peptides* **58**: 65–88

Johnson PWM, Joel SP, Love S, Butcher M, Pandian MR, Squires L, Wrigley PFM and Slevin ML (1993) Tumour markers for prediction of survival and monitoring of remission in small cell lung cancer. *Br J Cancer* **67**: 760–766

Kaplan EL and Meier P (1958) Nonparametric estimation from incomplete observations. *J Am Stat Assoc* **53**: 457–481

Konecki DS, Benedum UM, Gerdes H-H and Huttner WB (1987) The primary structure of human chromogranin A and pancreastatin. *J Biol Chem* **262**: 17026–17030

Mantel N (1966) Evaluation of survival data and two new rank order statistics arising in its consideration. *Cancer Chemother Rep* **50**: 163–170

Murray SS, Deaven LL, Burton DW, O'Connor DT, Mellon PL and Deftos LJ (1987) The gene for human chromogranin A (CgA) is located on chromosome 14. *Biochem Biophys Res Commun* **142**: 141–146

O'Connor DT and Deftos LJ (1987) How sensive and specific is measurement of plasma chromogranin A for the diagnosis of neuroendocrine neoplasia. *Ann N Y Acad Sci* **493**: 379–386

Østerlind K and Andersen PK (1986) Prognostic factors in small cell lung cancer: multivariate model based on 778 patients treated with chemotherapy with or without irradiation *Cancer Res* **46**: 4189–4194

Rosa P and Gerdes HH (1994) The granin protein family: markers for neuroendocrine cells and tools for the diagnosis of neuroendocrine tumors. *J Endocrinol Invest* **17**: 207–225

Sobol RE, O'Connor DT, Addison J, Suchocki K, Royston I and Deftos LJ (1986) Elevated serum chromogranin A concentrations in small-cell lung carcinoma. *Ann Intern Med* **105**: 698–700

Takiyyuddin MA, Cervenka JH, Pandian MR, Stuenkel CA, Neumman HPH and O'Connor DT (1990) Neuroendocrine sources of chromogranin-A in normal man: clues from selective stimulation of endocrine glands. *J Clin Endocrinol Metab* **71**: 360–369

Tarone RE (1975) Test for trend in life table analysis. *Biometrika* **62**: 679–682

*© 1999 Cancer Research Campaign*
